# Clinicopathological features and prognostic value of B7x expression in female reproductive system malignancies: A meta-analysis

**DOI:** 10.1097/MD.0000000000045150

**Published:** 2025-10-24

**Authors:** Quancang Men, Hang Su, Qian Yang, Xiaoru Qin

**Affiliations:** a Department of Thyroid and Breast Surgery, Hebei General Hospital, Shijiazhuang, China; b Department of Human Anatomy, Hebei Medical University, Shijiazhuang, China.

**Keywords:** B7x, malignant neoplasm of the reproductive system, meta-analysis, prognosis

## Abstract

**Background:**

B7x is overexpressed in female reproductive system malignancies. The aim of this study was to evaluate the predictive value of B7x expression in the clinicopathological characteristics and prognosis of female reproductive system malignancies.

**Methods:**

PubMed, Embase, Cochrane library, Web of Science, China National Knowledge Infrastructure, and Wanfang databases were searched for studies focused on the role of B7x expression in the clinicopathological features and prognosis of female breast cancer and malignant tumors of reproductive system, published up to April 2024. STATA 14.0 were used to perform the meta-analysis.

**Results:**

A total of 36 eligible studies involving 2451 women with malignancy of the reproductive system were included in this meta-analysis. The results showed that in terms of clinicopathological features, B7x was closely related to lymph node status (odds ratio [OR] = 2.80, 95% confidence interval [CI] = 1.54–5.11, *P* = .001), tumor differentiation (OR = 2.95, 95% CI = 1.91–4.57, *P* < .001), and FIGO stage (OR = 3.88, 95% CI = 3.04–4.94, *P* < .001) of female reproductive system malignant tumor patients. In terms of prognosis: B7x expression is strongly associated with shorter PFS in female reproductive system malignancies (HR = 1.30, 95% CI = 1.17–1.45, *P* < .001). B7x may be a new target for immunotherapy and a biomarker for predicting poor prognosis in female malignant tumors of reproductive system.

**Conclusion:**

In summary, B7x is closely associated with clinicopathological features and poor prognosis of malignant tumors of the female reproductive system.

## 1. Introduction

Malignant tumors of the reproductive system are the most common cause of cancer deaths in women. According to Global Cancer Statistics 2020, there will be approximately 8.2 million new cases of cancer in women in 2020, of which 16.50% will be malignant tumors of the reproductive system.^[1]^ With the increasing incidence of malignant tumors of the female reproductive system, and current treatments not significantly improving the prognosis of patients, there is an urgent need to find effective biomarkers and molecular targets for the early diagnosis and treatment of malignant tumors of the female reproductive system.

B7x was identified by Sica et al^[2]^ through bioinformatics in 2003, and Salceda et al^[3]^ found that B7x mRNA and protein overexpression in human breast and serous ovarian cancer further confirmed the existence of B7x, whose gene is mapped to the human 1p11.1 gene cluster. Studies have shown that B7x is expressed at high levels in female reproductive malignancies and breast cancer, while it is low in normal tissues.^[4–7]^ PD-1 and CD276, as homologous proteins of B7x, have been shown to be closely related to the occurrence and development of female breast cancer and reproductive system malignancies, and drugs targeting PD-1 and CD276 have shown good efficacy in cancer immunotherapy.^[8–15]^ Therefore, we believe that B7x may be a potential tumor marker and a potential immunotherapeutic target for the early diagnosis of female reproductive system malignancies.^[16]^ Nevertheless, the association of B7x with malignant tumors of the female reproductive system is somewhat controversial.

The purpose of this study was to evaluate the clinicopathological features and prognostic value of B7x in female malignant tumor of reproductive system by meta-analysis, and to provide evidence-based medical evidence for etiological research and clinical treatment.

## 2. Methods

### 2.1. Search strategy

We searched the following databases: PubMed, Cochrane Library, Embase, Web of Science, Wanfang databases, and China National Knowledge Infrastructure up to April 2024. Keywords used in the search include “Cervical Neoplasms,” “Ovarian Neoplasms,” “Endometrial Neoplasms,” “ B7x “, “ B7-H4,” “B7S1,” and corresponding Chinese terms. All information from the public databases is available and free for public, so the agreement of the medical ethics committee board was not necessary.

### 2.2. Inclusion and exclusion criteria

Inclusion criteria: the literature on the relationship between B7x and clinicopathologic features and/or prognosis of female reproductive system malignancies was included: hazard ratio (HR), odds ratio (OR), and 95% confidence interval (CI) or provide survival curves that we can use for prognostic meta-analyses; can refer to the full. Exclusion criteria: non-human experiments; review, conference abstract or case report; lack of relevant data; repeated publication.

### 2.3. Data extraction and quality evaluation

The extracted data includes: author, year, country, cancer type, sample size, sample type, detection method, exposure factors, and HR source. Meanwhile, we also extracted OR, HR, and 95% CI to analyze the prognostic value. For HR and 95% CI literature that do not directly provide OS, Engauge Digitizer version 4.1 is used to extract data according to the method described by Tierney et al.^[17]^ The quality of the included literature was assessed using the Newcastle-Ottawa Scale,^[18]^ with studies rated above 6 as high quality.

### 2.4. Statistical analysis

The OR and its 95% CI were used to assess the relationship between B7x expression and clinicopathological features in female malignant tumor of reproductive system. HR and its 95% CI were used to assess the relationship between B7x expression and prognosis in female malignant tumor of reproductive system. Statistically significant heterogeneity was defined as Cochran *Q* test *P* < .1 or Higgins *I*^2^ > 50%. If heterogeneity was observed, we used a random-effects model to reduce the effect of heterogeneity on results, otherwise, a fixed-effect model. If heterogeneity is significant, the source of heterogeneity is explored by sensitivity analysis and subgroup analysis. We used Begg and Egger tests to assess clinicopathological features and prognostic studies for publication bias, *P* < .05 was considered statistically significant. All statistical analyses are performed using STATA 14.0 software (StataCorp LLC, College Station).

## 3. Results

### 3.1. Key characteristics of the included studies

**Figure 1.**
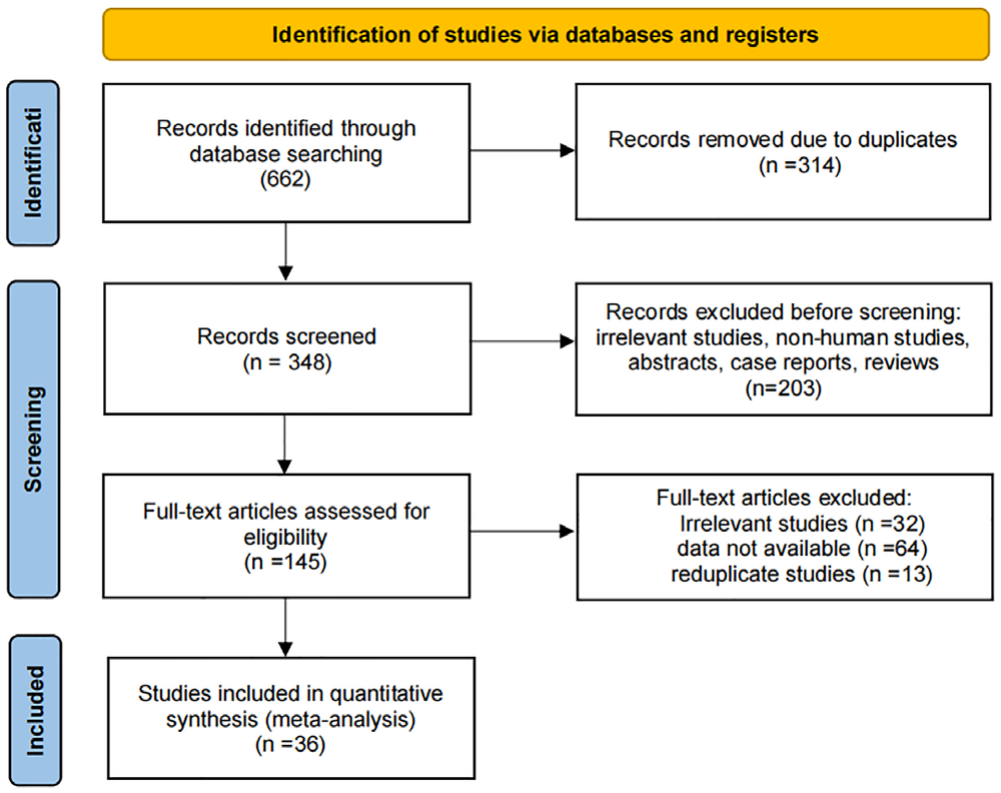
Flow diagram of the study selection process.

The process of screening the literature is shown in Figure 1, which resulted in the inclusion of 36 articles published between 2007 and 2023. A total of 2451 patients with female reproductive malignancies from 36 studies were included, and the characteristics of the included studies are shown in Table 1.

**Table 1:** Characteristics of included studies.

Author	Year	Country	Cancer type*	Sample size	Sample type	Detection method†	Exposure factors‡	HR source	NOS score
Chang J^[19]^	2009	China	OC	47	Tissue	IHC	1, 3, 4	–	7
Chen M^[20]^	2010	China	OC	63	Tissue	IHC	3, 4	–	6
Cui Z^[21]^	2019	China	OC	50	Tissue	IHC	2, 3	–	8
Du H^[22]^	2010	China	OC	30	Tissue	IHC	4	–	6
Geng W^[23]^	2011	China	CCA	67	Tissue	IHC	2	–	6
Guo L^[24]^	2016	China	OC	80	Tissue	IHC	1, 2, 3, 4	–	6
Han S^[25]^	2017	China	CCA	100	Tissue	IHC	2, 3	–	7
Hu X^[26]^	2021	China	CCA	112	Tissue	IHC	2, 3, 4	–	7
Huang C^[27]^	2016	China	CCA	108	Tissue	IHC	5	Estimated	8
Jiang Z^[28]^	2012	China	EC	55	Tissue	IHC	1, 3, 4	–	7
Kong F^[29]^	2009	China	OC	70	Tissue	IHC	2, 3, 4	–	6
Li H^[30]^	2016	China	OC	100	Tissue	IHC	2, 3, 4	–	7
Li N^[31]^	2016	China	EC	40	Tissue	IHC	1, 2, 4	–	6
Liao L^[32]^	2012	China	OC	86	Tissue	IHC	1, 2, 3, 4	–	6
Ling J^[33]^	2023	China	OC	46	Tissue	IHC	1, 3, 4	–	6
Liu C^[34]^	2019	China	CCA	50	Tissue	IHC	2	–	7
Liu H^[35]^	2021	China	CCA	109	Tissue	IHC	2, 3	–	8
Liu W^[36]^	2013	Japan	CCA	102	Tissue	IHC	1, 2	–	6
Liu Y^[37]^	2014	China	EC	50	Tissue	IHC	2, 3, 4	–	7
Liu Y^[38]^	2013	China	OC	70	Tissue	IHC	4	–	6
lv Y^[39]^	2009	China	EC	50	Tissue	IHC	3, 4	–	6
Ma D^[40]^	2018	China	CCA	40	Tissue	IHC	1, 2, 3	–	7
Ma Z^[41]^	2011	China	OC	35	Tissue	IHC	1, 3, 4	–	7
Niu N^[42]^	2021	USA	OC	48	Tissue	IHC	4	–	6
Simon I^[43]^	2007	USA	OC	251	Serum	ELISA	4, 5	Reported	7
Sun S^[44]^	2017	China	OC	30	Tissue	IHC	1, 2, 4	–	6
Teng Y^[45]^	2009	China	EC	48	Tissue	IHC	3, 4	–	6
Wang Y^[46]^	2009	China	OC	40	Tissue	IHC	2, 3, 4, 5	Estimated	6
Wang Y^[47]^	2011	China	OC	50	Tissue	IHC	2, 4	–	6
Xu M^[48]^	2016	China	OC	112	Tissue	IHC	2, 3, 4, 5	Reported	7
Zhang H^[49]^	2014	China	OC	45	Tissue	IHC	1, 2, 4	–	6
Zhang L^[50]^	2010	China	OC	40	Tissue	IHC	2, 3, 4	–	6
Zhang X^[51]^	2018	China	OC	58	Tissue	IHC	3, 4	–	6
Zhang Y^[52]^	2013	China	OC	66	Tissue	IHC	1, 2, 3, 4	–	7
Zhao Y^[53]^	2015	China	OC	52	Tissue	IHC	2, 3	–	7
Zhong G^[54]^	2010	China	OC	51	Tissue	IHC	1, 2, 3, 4	–	7

NOS = Newcastle-Ottawa Scale.

* Cancer type: CCA = cervical cancer; EC = endometrial cancer; OC = ovarian cancer.

† Detection method: IHC = immunohistochemistry; ELISA = enzyme linked immunosorbent assay.

‡ Exposure factors: 1. age (≥50 vs <50); 1. lymph node status (N1–3 vs N0); 2. tumor differentiation (well and moderate vs poorly); 3. FIGO stage (III + IV vs I + II); 5. PFS.

### 3.2. Association between B7x and clinicopathological features of malignant tumors of the female reproductive system

#### 3.2.1. Age

**Figure 2.**
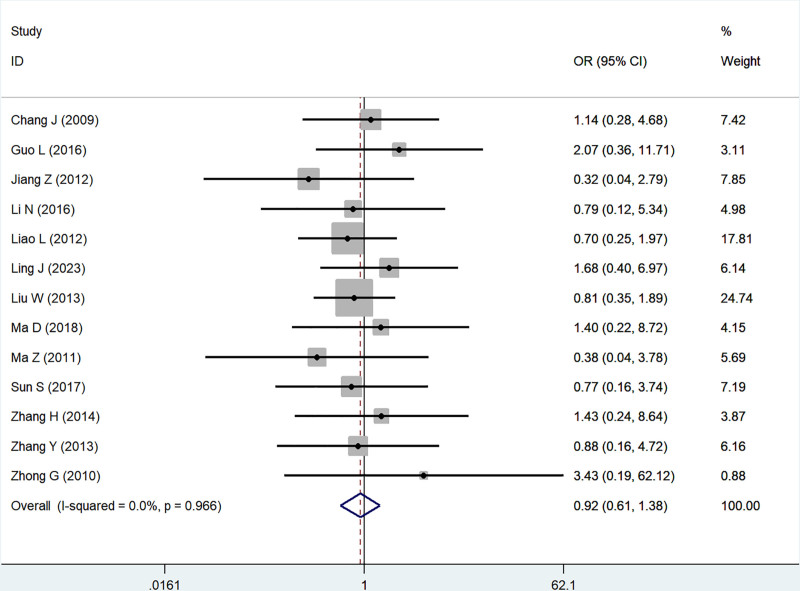
Forest plot of association between B7x expression and age in female reproductive system malignancies patients based on a fixed-effect model (*P* = .68).

Thirteen studies reported the association of B7x with age (≥50 vs <50), demonstrating no heterogeneity by testing (*I*^2^ = 0.0%, *P* = .97), and using a fixed-effect model, the results showed that B7x was not associated with age (OR = 0.92, 95% CI = 0.61–1.38, *P* = .68) (Fig. 2).

#### 3.2.2. Lymph statue

**Figure 3.**
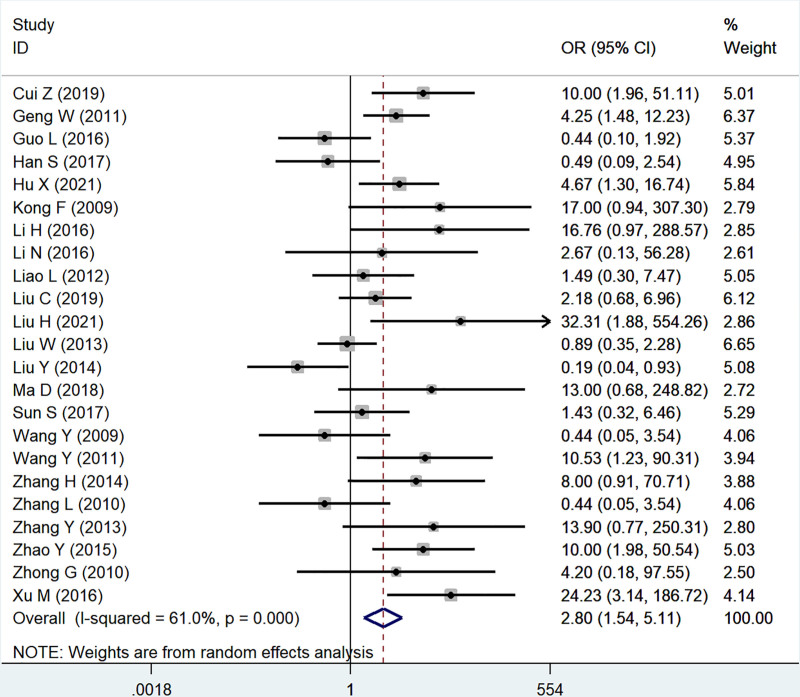
Forest plot of association between B7x expression and lymph statue in female reproductive system malignancies patients based on a random-effect model (*P* = .001).

Twenty-three studies reported the association of B7x with lymph statue (N1–3 vs N0). Significant heterogeneity was proved by testing (*I*^2^ = 61.0%, *P* < .001), and using a random-effect model, the results showed that B7x was associated with lymph statue (OR = 2.80, 95% CI = 1.54–5.11, *P* = .001) (Fig. 3).

#### 3.2.3. Tumor differentiation

**Figure 4.**
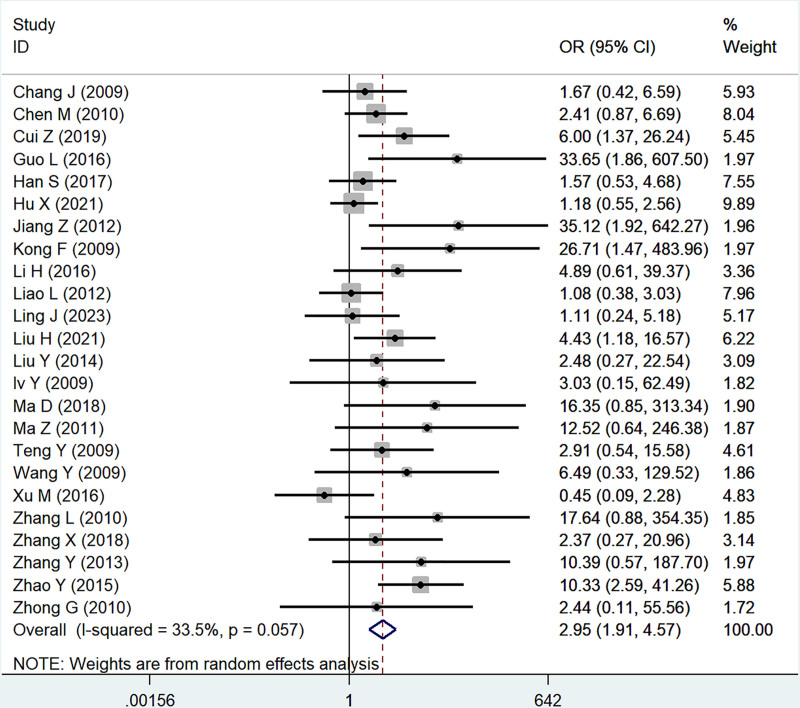
Forest plot of association between B7x expression and tumor differentiation in female reproductive system malignancies patients based on a random-effect model (*P* < .001).

Twenty-four studies reported the association of B7x with tumor differentiation (well and moderate vs poorly), significant heterogeneity was proved by testing (*I*^2^ = 33.5%, *P* = .06), and using a random-effect model, the results showed that B7x was associated with tumor differentiation (OR = 2.95, 95% CI = 1.91–4.57, *P* < .001) (Fig. 4).

#### 3.2.4. FIGO stage

**Figure 5.**
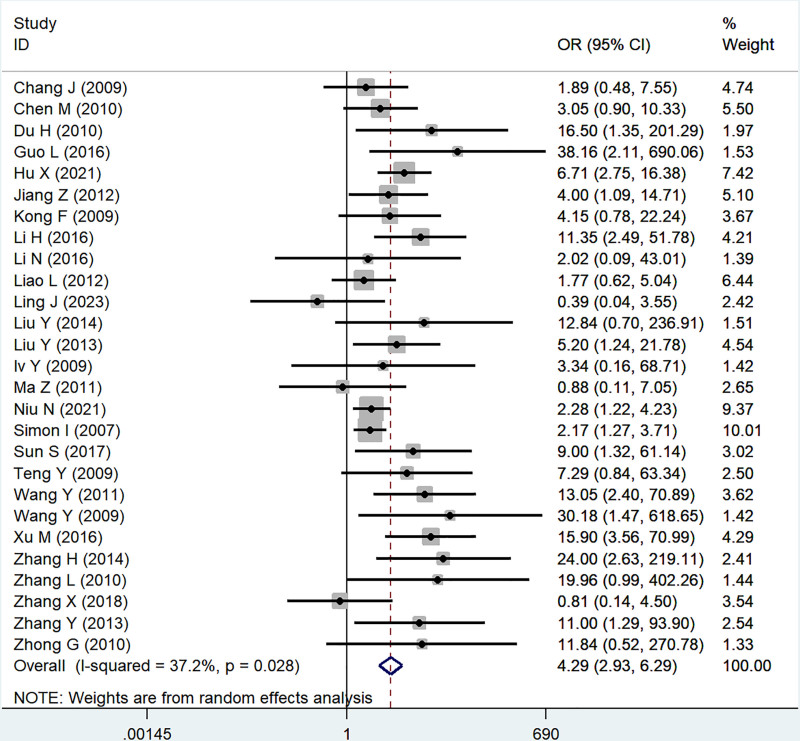
Forest plot of association between B7x expression and FIGO stage in female reproductive system malignancies patients based on a random-effect model (*P* < .001).

Twenty-seven studies reported a correlation between B7x and FIGO stage (III + IV vs I + II), significant heterogeneity was proved by testing (*I*^2^ = 37.2%, *P* = .03), and using a random-effect model, the results showed that B7x was associated with FIGO stage (OR = 4.29, 95% CI = 2.93–6.29, *P* < .001) (Fig. 5).

#### 3.2.5. Sensitivity analysis and subgroup analysis

**Figure 6.**
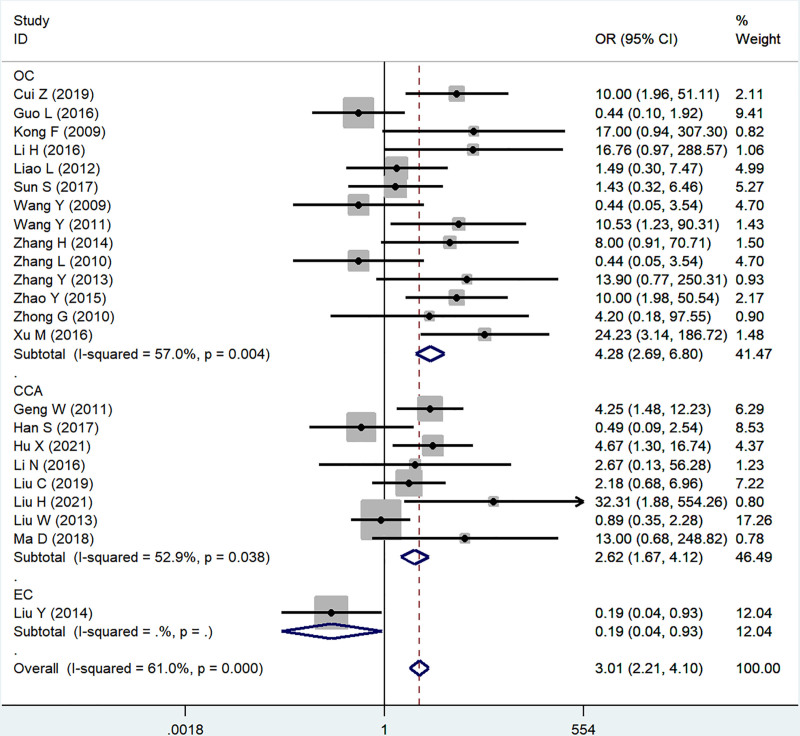
Subgroup analysis was performed on the relationship between B7x expression level and lymph statue of patients with female reproductive system malignancy based on disease type.

**Figure 7.**
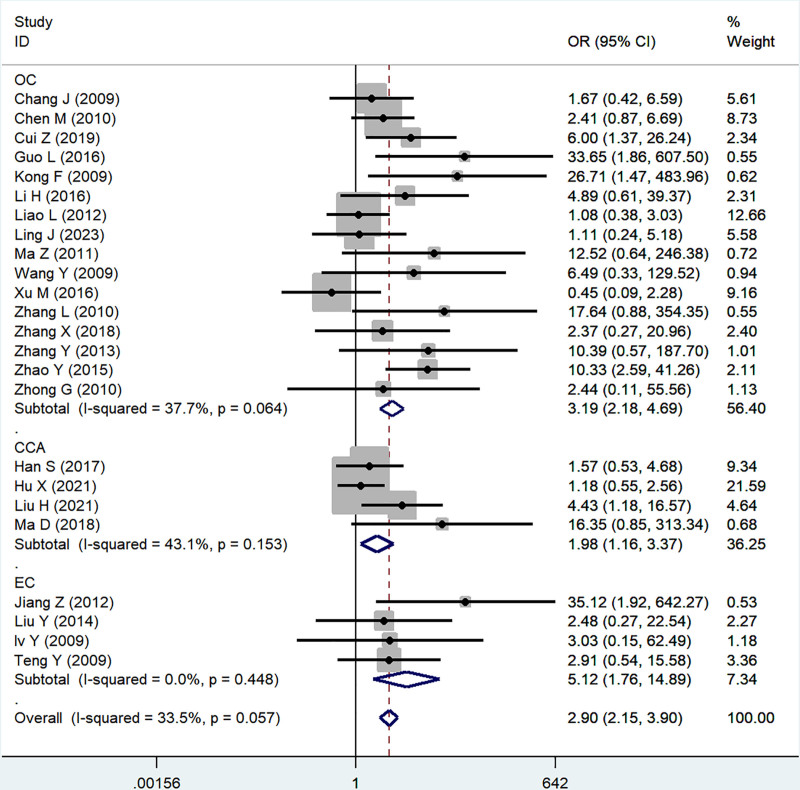
Subgroup analysis was performed on the relationship between B7x expression level and tumor differentiation of patients with female reproductive system malignancy based on disease type.

**Figure 8.**
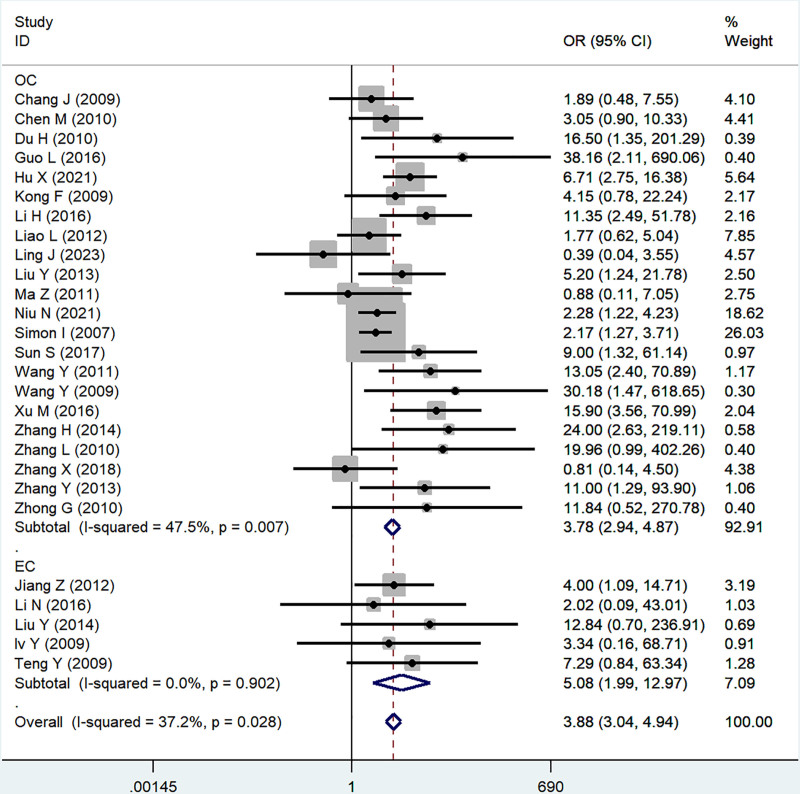
Subgroup analysis was performed on the relationship between B7x expression level and FIGO stage of patients with female reproductive system malignancy based on disease type.

In the study of B7x associated with lymph node status, tumor differentiation and FIGO stage in female reproductive system malignancies, we performed subgroup analyses based on disease type due to significant heterogeneity, and the results showed no significant difference between subgroups (Figs. 6–8). We also tested the effect of individual studies on outcomes by sensitivity analyses, and no significant differences were found after excluding each article in turn, indicating the stability of the results of the meta-analysis.

### 3.3. Association of B7x with prognosis of female reproductive system malignancies

**Figure 9.**
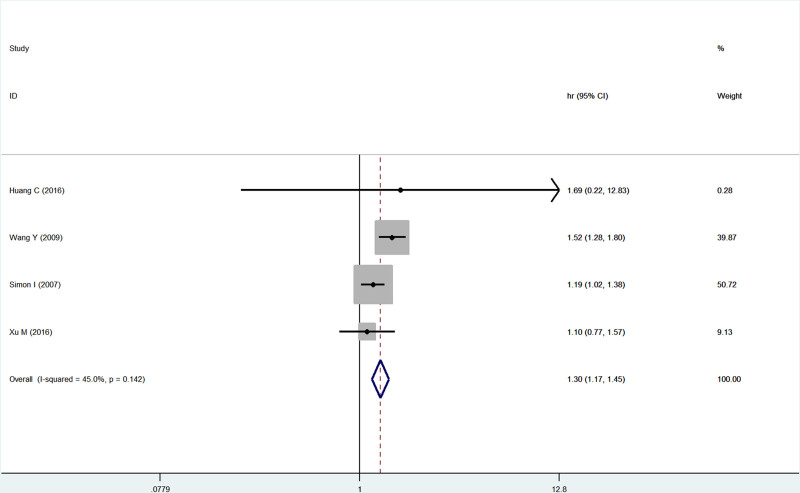
Forest plot of association between B7x expression and PFS in female reproductive system malignancies patients based on a fixed-effect model (*P* < .001).

Four studies reported the association of B7x with PFS in patients with female reproductive system malignancies, demonstrating no heterogeneity by testing (*I*^2^ = 45.0%, *P* = .14), and using a fixed-effect model, the study showed that B7x was strongly associated with shorter PFS in patients with malignancies of the female reproductive system (HR = 1.30, 95% CI = 1.17–1.45, *P* < .001) (Fig. 9).

### 3.4. Publication bias

Begg funnel plots and Egger tests were used to assess publication bias of clinicopathological features and prognostic studies. The symmetry of the Begg funnel plot and the results of the Egger linear regression test showed that there may be some publication bias in the study on tumor differentiation and FIGO stage of female reproductive system malignant tumors, while there was no publication bias in other indicators. The summary results are shown in Table 2. Therefore, the regression correction was performed by cut-and-compensation method, and after correction, it was found that it was necessary to add 9 and 8 articles, respectively, to ensure the symmetry of the funnel and eliminate publication bias.

**Table 2:** Summary of *P* values of the association between Begg and Egger and the clinicopathological features of female reproductive system malignancies.

	Age	Lymph statue	Tumor differentiation	FIGO stage	PFS
Begg	0.86	0.20	0.04	0.17	0.73
Egger	0.69	0.08	0.001	0.01	0.96

## 4. Discussion

In recent years, with the increasing incidence of malignant tumor of female reproductive system, its early diagnosis and treatment have become more and more important. Due to the lack of effective early detection methods, the mortality rate of malignant tumors of the female reproductive system is high.^[55]^ Therefore, it is necessary to search for accurate and reliable tumor markers for the early diagnosis and targeted therapy of female reproductive system malignant tumors.

B7x is overexpressed in female breast cancer and malignant tumors of the reproductive system.^[4–7]^ It can participate in cancer immune escape by inhibiting T cell proliferation, inducing cell cycle arrest, reducing the secretion of cytokines IL-2, TNF-α, and INF-γ, promoting the development of regulatory T cells and other mechanisms.^[56,57]^ In addition, it can also be involved in tumor cell proliferation and metastasis by activating signal transduction pathways, inducing epithelial mesenchymal transformation, and increasing chemotherapy resistance, among other non-immune pathways.^[3,58,59]^ Therefore, B7x is likely to be a potential tumor marker and a potential immunotherapeutic target for the early diagnosis of malignant tumors of the female reproductive system. At present, there are few studies on the relationship between B7x and malignant tumors of female reproductive system. Ye Y et al^[60]^ conducted a meta-analysis of 10 studies involving 1045 patients, and the results showed that B7x was associated with poor prognosis of ovarian cancer, but not with clinicopathological characteristics of patients.

Our latest study comprehensively analyzed the clinicopathological features and prognostic value of B7x in female reproductive system malignancies, including 36 eligible studies involving 2451 female reproductive system malignancies. In terms of the association of B7x with clinicopathological features of female reproductive system malignancies, our meta-analysis showed that B7x was strongly associated with lymph node metastasis, tumor differentiation, and FIGO stage, regardless of patient age. This is contrary to the conclusion of the previous study by Ye Y et al,^[60]^ but we included more studies, so our conclusion is more convincing. In terms of the correlation between B7x and the prognosis of female reproductive system malignant tumors, the results of our meta-analysis showed that B7x was significantly correlated with shorter PFS, which was consistent with the conclusions of previous studies. Therefore, B7x may be a reliable biomarker for the early diagnosis of malignant tumors of the female reproductive system and a molecular target for immunotherapy.

To the best of our knowledge, this meta-analysis is the largest sample study to date evaluating the association of B7x with malignancies of the female reproductive system. However, this meta-analysis has certain limitations. First of all, there was significant heterogeneity in some studies, but no major sources of heterogeneity were found through subgroup analysis and sensitivity analysis, so it is necessary to further expand the sample size to find potential sources of heterogeneity. Secondly, most of the literatures included in this study are single-factor analyses. If multi-factor analysis is adopted, it is necessary to further collect data, improve statistical methods and improve literature quality. In addition, some data and their 95% CI are obtained indirectly through software, and there may be some deviation between them and the real survival data, which reduces the reliability of the results. Finally, this paper includes the studies of many countries, and the pertinence is not strong. In the later stage, the number of articles can be increased to carry out subgroup analysis and study the gene expression of people in different regions.

## 5. Conclusion

In summary, B7x is closely associated with clinicopathological features and poor prognosis of malignant tumors of the female reproductive system. B7x may become a new target for immunotherapy of malignant tumors of the female reproductive system and a biomarker for predicting the poor prognosis of malignant tumors of the female reproductive system. In the future, multicenter and prospective studies need to be carried out to further confirm the association between B7x and the clinicopathological characteristics and prognosis of malignant tumors of the female reproductive system.

## Author contributions

**Conceptualization:** Quancang Men, Hang Su.

**Data curation:** Quancang Men, Hang Su, Qian Yang, Xiaoru Qin.

**Formal analysis:** Quancang Men, Hang Su, Xiaoru Qin.

**Funding acquisition:** Quancang Men, Hang Su.

**Investigation:** Quancang Men, Hang Su, Qian Yang.

**Methodology:** Quancang Men, Hang Su.

**Project administration:** Quancang Men, Hang Su.

**Resources:** Quancang Men, Hang Su.

**Software:** Quancang Men, Hang Su.

**Supervision:** Quancang Men, Hang Su.

**Validation:** Quancang Men, Hang Su.

**Visualization:** Quancang Men, Hang Su.

**Writing – original draft:** Hang Su.

**Writing – review & editing:** Hang Su.
